# Heavy Metal Accumulation in Cattle from Western Pará: Human Health Risk Assessment

**DOI:** 10.3390/toxics13090740

**Published:** 2025-08-31

**Authors:** Antonio Humberto Hamad Minervino, Osvaldo Gato Nunes Neto, Fábio Edir Amaral Albuquerque, Kelly Cristiny Gomes da Paixão Albuquerque, Francisco Flávio Vieira de Assis, Rejane Santos Sousa, Raimundo Alves Barrêto Júnior, Marta López-Alonso, Marta Miranda

**Affiliations:** 1 Laboratory of Animal Health, LARSANA, Federal University of Western Pará, UFOPA, Rua Vera Paz, s/n, Salé, Santarém 68040-255, PA, Brazil; vet.osvaldo@gmail.com (O.G.N.N.); fabio.edir@yahoo.com.br (F.E.A.A.); ff.assis@outlook.com (F.F.V.d.A.); 2 Ph.D. Program in Society, Nature and Development, PPGSND, Federal University of Western Pará, UFOPA, Rua Vera Paz, s/n, Salé, Santarém 68040-255, PA, Brazil; 3 Centro Universitário da Amazônia, UNAMA, Rua Rosa Vermelha, 335—Aeroporto Velho, Santarém 68010-200, PA, Brazil; 4 Ph.D. Program in Animal Health in the Amazon, PPGSAAM, Federal University of Pará, UFPA, University Avenue, s/n, Jaderlândia, Castanhal 68746-360, PA, Brazil; kellycpaixao@yahoo.com.br; 5 Instituto de Estudos do Trópico Úmido, IETU, Universidade Federal do Sul e Sudeste do Pará, UNIFESSPA, Xinguara 68557-335, PA, Brazil; rejane.sousa@unifesspa.edu.br; 6 Department of Animal Science, Federal Rural University of the Semiarid Region, UFERSA, Mossoró 59625-900, RN, Brazil; barreto@ufersa.edu.br; 7 Department of Animal Pathology, Veterinary Faculty, University of Santiago de Compostela, 27002 Lugo, Spain; 8 Department of Anatomy, Animal Production and Clinical Veterinary Sciences, Veterinary Faculty, University of Santiago de Compostela, 27002 Lugo, Spain

**Keywords:** toxic metals, mercury, bovine, environmental pollution, target hazard quotient

## Abstract

Western Pará, northern Brazil, is a significant region for mineral exploration, leading to the deposition of potentially toxic elements in soils and water basins. This study evaluated concentrations of mercury (Hg), lead (Pb), cadmium (Cd), and arsenic (As) in cattle muscle tissue from three municipalities: Oriximiná, Itaituba, and Monte Alegre. Metal concentrations were determined using inductively coupled plasma mass spectrometry (ICP-MS). The estimated daily intake (EDI) of toxic metals via beef consumption (71 g/person/day) was below oral reference doses values (RfDo). Target hazard quotient (THQ) and total THQ (TTHQ) values for all metals were below 1, indicating no significant non-carcinogenic health risk. Monte Alegre exhibited the highest THQ for As and Pb, Oriximiná for Cd, and Itaituba for Hg. Although the overall assessment suggests low risk, elevated Hg concentrations were detected in 10% of the samples, with at least one animal from each municipality exceeding the European Union maximum residue limit (0.01 mg/kg). These findings indicate localized contamination and potential mercury bioaccumulation. Given the rising anthropogenic activities (such as mining and deforestation), continued monitoring of heavy metal levels in animal tissues is recommended to ensure long-term food safety and public health.

## 1. Introduction

The Amazon region is a recognized hotspot for mercury (Hg) contamination, with extensive research focused on bioaccumulation in fish and health risks to local populations [1,2,3]. The Tapajós River basin in Pará State is particularly affected due to intensive gold mining activities that utilize mercury, contributing to widespread environmental contamination, and human exposure [4]. In addition to the well-known contamination due to gold mining, Hg is present in large amounts in Amazon soils and can reach aquatic environments due other anthropogenic activities that cause soil erosion, such as mining, deforestation, and agriculture expansion [5]. Another Hg source is wildfires, which are estimated to emit 13% of the atmospheric mercury in Brazil, which is equivalent to 8.7 tons per year [6,7].

While mercury is a major concern, other toxic elements such as arsenic (As), cadmium (Cd), and lead (Pb) [4] are known to be present in the region, whether due to anthropic or natural action [8]. As contamination is described in Santana, Amapá, where this metal is associated with manganese ores exploited in the last 50 years [9]. Arsenic concentration is high in the Andean rivers (Solimões and Madeira), with values higher than 0.8 mg/m^3^ of arsenic in the Amazon River (Óbidos), indicating that about 0.5 tons of As flows to the ocean daily (200 tons annually) [10].

The contamination by Pb in the Brazilian Amazon, especially in the state of Pará, was studied in soil [11], sediment [12], water [13] and aquatic animals (fish and shrimp) [3,14]. The high concentrations of Pb found in riverside populations from Brazilian Amazon reveal the need for constant monitoring of exposure to this metal, with significant consequences for public health [15,16,17]. In addition to the Pb natural presence, the use of lead as a component of paints, batteries, varnishes, greases, waxes, and other products that, when discarded, can pollute the environment [16,18].

Anthropogenic sources of Cd emissions, such as industrial activities and fossil fuel combustion, often exceed natural sources like volcanic activity and soil erosion [19,20]. Cadmium and Cd compounds have negligible vapor pressures, but can exist in the air, as suspended particles, in industrial emissions, fuel combustion or in soil erosion [20]. In aquatic systems, rivers are estimated to carry 30,000 tons of Cd annually to the oceans through weathering and erosion processes. Additionally, atmospheric deposition contributes 4000 to 13,000 tons of cadmium to aquatic environments worldwide from both natural and anthropogenic sources [21,22].

Although Hg contamination in fish is largely reported in Amazon region, few studies have investigated heavy metal accumulation in cattle. To our knowledge, no human health risk assessments have evaluated toxic metal contamination in cattle, despite of frequent heavy metals reports in the Amazon environment and their potential effects on human health, and beef being the most consumed meat in Brazil [22]. 

Cattle ranching in the western Pará (mainly in the lower Amazon mesoregion) may be prone to heavy metal contamination due to a combination of factors. Pastures with natural forage species in wetland areas are annually flooded in the rainy season [23,24,25]. Seasonal flooding contributes to natural fertilization of floodplains involving the accumulation of mineral and organic colloids in low-lying areas along the Amazon River and its tributaries [26,27,28]. In addition, seasonal flooding leads to changes in the bioavailability of toxic elements, which can be deposited in the soil [29,30]. During floods sediments adsorb heavy metals, extending the range and transfer of metals, resulting in the accumulation of these toxic metals in native plant and animal tissues, entering the food chain [31,32]. This scenario is further worsened in the region due large bauxite mining projects and artisanal gold mining [3,8]. These mining activities, along with agricultural activities, mainly corn and soybean cultivation, have led to intense deforestation [33,34]. These activities often lead to environmental contamination involving potentially toxic elements which can have harmful effects on organisms if consumed in large quantities [35,36]. 

The combination of flooding phenomena and socio-economic activities in the region intensifies the risks of environmental contamination and the related risk to the health of ecosystems and local communities. Despite this, the estimation of the carcinogenic and non-carcinogenic risk of metal contamination from meat consumption has not been studied in the Amazon region. Thus, we aimed to determine the presence of toxic metals in bovine muscle in different cities in the western region of Pará subject to different types of metal contamination and to perform a risk analysis to human health of the consumption of beef from animals slaughtered in the region. 

## 2. Materials and Methods

### 2.1. Study Site and Sample Collection

**Figure 1:**
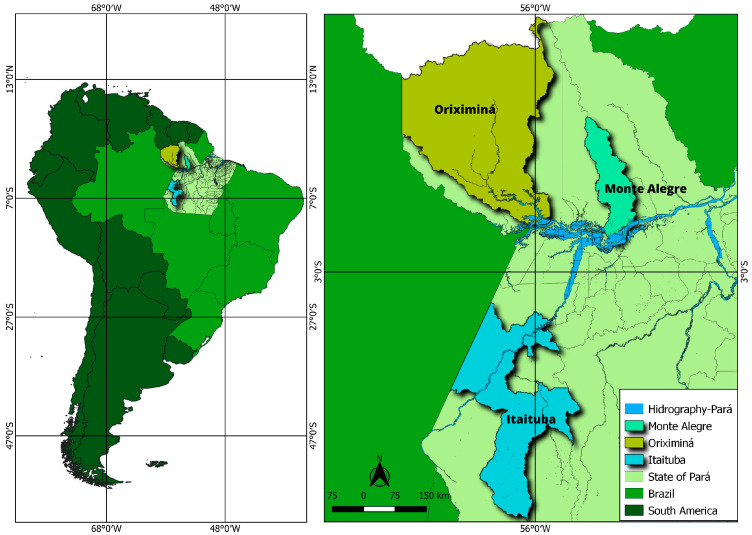
Identification and location of sampling sites in the western part of the state of Pará.

Samples were taken from three municipalities located in western Pará, in the lower Amazon (Figure 1). Two were selected for their intense mineral exploitation: Itaituba, where the largest small-scale gold mines are located; Oriximiná, a region where bauxite has been exploited since 1969; and Monte Alegre, which has no mining activity, but is under pressure from agricultural activity, mainly monocultures such as soya and maize. In each municipality, ten samples of muscle tissue from the tail (medial dorsal sacrocaudal muscle), weighing 100 g each, were collected from different butchers, totaling 30 samples, which were then stored at an average temperature of 4 °C, and to the laboratory and frozen at −20 °C until analysis. Although we do not have precise information about animal origin, we sampled from different butcher shops on different days, choosing specific types of butcher shops, to ensure that each sampled animal came from a different farm within the municipality. Previous visits to the butcher were performed to obtain information with the owners about the source of the meat, and the sampling scheme was based on this information to spread our limited sample size across the greatest number of farms possible.

### 2.2. Sample Preparation

Once in the laboratory, each specimen was measured and weighed. Subsamples of approximately 1 g were accurately weighed and digested in a mixture of 5 mL of concentrated nitric acid (TMA, Hiperpure, PanReac, Castellar del Vallès, Spain) and 3 mL of 30% *w*/*v* hydrogen peroxide (PanReac, Spain) in a microwave-assisted digestion system (Ethos Plus; Milestone, Sorisole, Italy). Digested samples were transferred to polypropylene sample tubes and diluted to 15 mL with ultrapure water according to previously described procedures and conditions [37].

### 2.3. Toxic Element Analysis

The concentrations of the non-essential elements arsenic (As), cadmium (Cd), mercury (Hg), and lead (Pb) in the digested samples were determined by inductively coupled plasma mass spectrometry (Agilent 7900 × ICP-MS system; Agilent Technologies, Tokyo, Japan). A detailed description of the analytical conditions is provided elsewhere [9,38]. Analytical quality control was applied throughout this study. Blank samples were processed at the same time as test samples, and the values obtained were subtracted from sample readings for the calculation of the final values. The limits of detection (LODs) were calculated as three times the standard deviation of the reagent blanks. In all cases, the obtained LODs were low enough to determine all metals at the usual levels in the studied samples [3]. The accuracy of the determination was assessed by comparison with the analytical recovery of certified reference materials (bovine liver, BCR-185R: Institute for Reference Materials and Measurements, Geel, Belgium) used to validate the ICP-MS measurements [39]. The good agreement between the measured and the certified values demonstrated the high accuracy of the method (recoveries between 88.5 and 106.9%). The precision of the analytical method, calculated as the relative standard deviation (RSD) of 10 different extractions of the same sample, ranged between 5.7 and 9.4%.

### 2.4. Human Health Risk Assessment

To assess the risk to human health associated with the consumption of beef contaminated with toxic metals various methods were employed, including the estimated daily intake (EDI), the hazard quotient (HQ), target hazard quotients (THQ), and the total target hazard quotient (TTHQ) [38,40,41,42,43]).

To determine the risk of heavy metals from beef consumption, the calculations were based on: an average adult body weight of 70 kg [44], and an average per capita consumption rate of beef in Brazil of 26 kg per year, i.e., 71 g/person/day [45]. The following indices were calculated according to their respective equations:

#### 2.4.1. Estimated Daily Intake (EDI)

To calculate the EDI, the average metal content from each city was multiplied by the specific consumption rate [40,42,43]. EDI is expressed in mg/kg of body weight per day [46] and was calculated using Equation (1):

(1) $$EDI=\frac{C\times Ccons}{Bw}$$

where C is the concentration of heavy metals in bovine muscle (mg/kg fresh weight), C_cons_ is the average daily consumption of beef in the region and the national daily intake rate (71 g/day), and Bw represents the body weight of adults (70 kg). 

#### 2.4.2. Non-Carcinogenic Risk

The hazard quotients calculate the ratio between the exposure dose and the reference dose (RfD), representing the risk of non-cancerous effects. An HQ < 1 indicate that the exposure level is below the reference dose, thus the daily exposure at this level is unlikely to have negative effects during a person’s lifetime, and therefore the hazard can be considered negligible. Conversely, an HQ > 1.0 suggests potential health risks, as exposure exceeds the reference concentration, and consumption of beef may constitute a health hazard for the consumer [47,48,49]. The RfDs defined for As, Cd, Hg, and Pb are AS 0.0003, Cd 0.001, Hg 0.005, and Pb 0.004 mg/kg Bw/day [42].

##### Determination of Hazard Quotient (HQ)

The HQ relates the exposure dose a to a toxicological end point: the reference dose RfD [49]

(2) $$HQ=\frac{EDI}{RfDo}$$

where RfD is the oral reference dose for a given element (As 0.0003, Cd 0.001, Hg 0.005, and Pb 0.004 mg/kg Bw/day).

##### Determination of Target Hazard Quotient (THQ)

The model for estimating THQ was determined by the following equation [42,45]:

(3) $$THQ=\frac{EFr\times EDtot\times FIR\times C}{RfDo\times Bw\times ATn}{\times 10}^{-3}\;$$

where EFr is the frequency of exposure (365 days/year); Ed_tot_ is the duration of exposure (70 years); FIR is the rate of food intake (g/day), while 10^−3^ is the unit conversion factor; C is the heavy metal concentration in bovine muscle (mg/kg fresh weight); RfD is the oral reference dose for a given element (As 0.0003, Cd 0.001, Hg 0.005, and Pb 0.004 mg/kg Bw/day); Bw is the average adult body weight (70 kg); and ATn is the average exposure time for non-carcinogens (365 days/year × number of years of exposure, assuming 70 years) [49].

##### Determination of Total Target Hazard Quotient (TTHQ)

In this study, the total THQ was expressed as the arithmetic sum of the individual THQ values for each of the metals analyzed (As, Cd, Hg, and Pb) [40,42,43]:


TTHQ = THQ (As) + THQ (Cd) + THQ (Hg) + THQ (Pb)
(4)

#### 2.4.3. Carninogenic Risk

##### Cancer Risk (CR) for Arsenic e Cadmium

The concentrations of potential carcinogens (As and Cd) in the bovine muscle tissue were used to estimate the risk of cancer development The ingestion dose exhibits a directly proportional relationship with the quantity of carcinogen ingested, with its effects being quantified through carcinogenic risk (CR) assessment. The CR was calculated for each metal using the following equation:

(5) CR=CSFo × EDI

where CSFo represents the carcinogenic slope factor or lifetime possibilities of having cancer, with a CSFo of 1.5 and 0.38 for As [50] and Cd, mg/kg/day, respectively [51,52]. Carcinogenic risk values of 1.0 × 10^−4^ and multiple-element (MCR) < 1.0 ×10^−4^ are tolerated and do not enhance the risk of having cancer for a lifetime [53].

##### Total Cancer Risk (TCR)

For the assessment of combined exposure to As and Cd, the TCR was defined as the arithmetic sum of the individual CR values for each analyzed metal, using Equation (6)

(6) $$MCR=\sum_{i=1}^{n}CR$$

### 2.5. Statistical Analysis

Principal Component Analysis (PCA) was performed using Canoco software (version 4.5).

## 3. Results

### 3.1. Concentration of Toxic Metals in the Muscle of Cattle

The concentrations of toxic metals in cattle muscle from the municipalities of Western Pará are presented in Table 1. According to the results, the concentration range of toxic elements expressed in mg/kg of wet weight was As: 0.0003–0.059; Cd: 0.0004–0.037; Hg: 0.001–0.035; and Pb: 0.011–0.242.

### 3.2. Principal Component Analysis (PCA) of the Distribution of Toxic Metals

**Figure 2:**
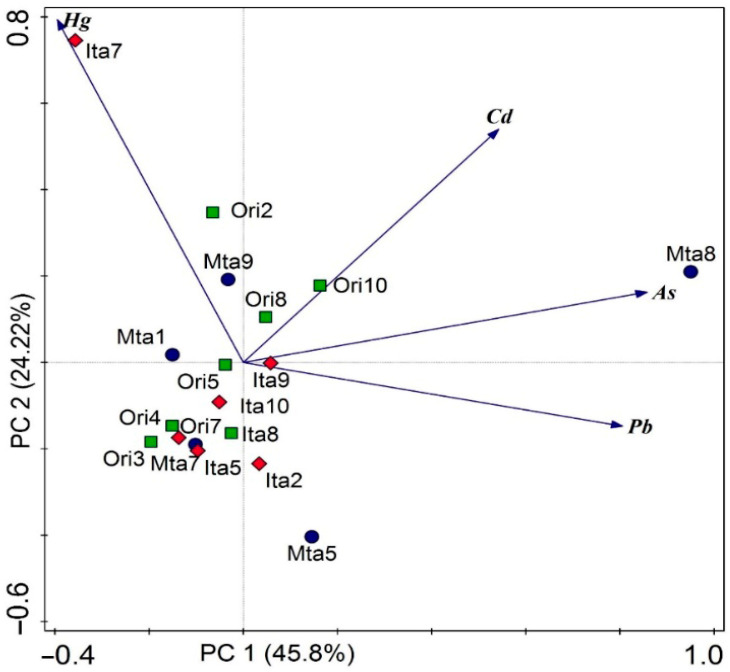
Ordering diagram of the analysis of principal components of heavy metals sampled from the muscles of cattle from three large centers in western Pará. Legend: Ori—Oriximiná; Mta—Monte Alegre; Ita—Itaituba.

The PCA was used to study the relationships between the variables. The results of the loads of the different bioaccumulations of the four metals in the muscle samples of cattle are presented in the reduced space of the two main components (Figure 2). The first axis (PC1) represents a gradient of the metals As and Pb, explaining 45.8% of the variability of the data. The second axis (PC2) represents a Hg gradient, explaining 24.22% of the variability of the data sampled from the cities considered in this study. The PCA loadings (Table 2) indicate the primary variables contributing significantly to each axis. For the PC1, As exhibits the highest contribution to this gradient, followed by Pb. For the PC2, the greatest contribution is observed for Pb. It is noteworthy that the highest concentrations of As were recorded in the sample collected from Monte Alegre, which lacks mineral activity such as the gold mining in Itaituba or bauxite mining in Oriximiná, while the highest levels of Hg were found in samples from Itaituba. PCA revealed that the first component (PC1), accounting for 45.8% of the variance, is dominated by loadings of As, Pb, and Cd, with Hg showing a negative loading. The second component (PC2), explaining 22.2% of the variance, is led by Hg and Cd, displaying a negative loading, and As near zero.

### 3.3. Human Health Risk Assessment

In this study, the results of the calculation of the EDI (Table 3), which represents the daily intake of toxic metals through the daily consumption of beef (71 g per person), in general, indicated that the concentrations of metals were below the reference values (RfD).

The results of the HQ (Table 4) indicated that all toxic metals had a value lower than 1 and that the adverse effects of an ingestion are unlikely.

The results of the analysis of the target risk quotient (THQ) and the data of exposure to multiple contaminants (TTHQ) are presented in Table 5. According to Table 6, the values of the four toxic elements were not associated with risks due to isolated exposure (THQ < 1). However, the results of TTHQ were analogous to the values found in the THQ calculations.

All individual CR and TCR values (Table 6) remained below the acceptable threshold (1.0 × 10^−4^), indicating that exposure to these metals through bovine meat consumption does not pose a significant carcinogenic risk to the local population. Arsenic (As) was identified as the primary contributor to the risk, reflecting its higher carcinogenic potency.

## 4. Discussion

The mean concentrations of heavy metals in cattle muscle were below the maximum limits established by the European Union (MRL EU) [54] and by Brazil (MRL Brazil) [55], in all the three municipalities studied. Despite the documented scenario of environmental contamination in the Amazon region, especially from mineral exploration, our results contrast with the high levels of toxic metals reported in fish species in the region [3,8]. We suggest that the metal concentrations found may not reveal a bioaccumulative status in cattle muscle due to the average slaughter time, which in the Amazon region range from 2.5 to 3 years, which may not allow sufficient time for significant bioaccumulation. Further studies focusing in older cattle, such as discarded females (>8 years) may show different pattern of metal exposure. 

In order to better understand spatial pattens and environmental drivers underlying metal accumulation in cattle, a Principal Component Analysis (PCA) was conducted, which explained 70.02% of the total variance in heavy metal (As, Cd, Pb and Hg) bioaccumulation across the sampled animals. This multivariate approach revealed two principal components with significant environmental relevance and allowed the samples to be spatially differentiated based on their origin [56]. The first principal component (PC1) showed strong loadings for As and Pb, indicating the predominance of these elements in samples from Monte Alegre and Oriximiná. These municipalities are influenced by the hydrosedimentary dynamics of the Amazon River, the main source of suspended sediments in the region [57], which act as natural carriers of trace elements, in-creasing their bioavailability in forage grasses consumed by cattle [57,58]. 

Naturally occurring heavy metals typically appear as trace components in detrital minerals, whereas anthropogenic metals, derived from activities such as mining, deforestation, agriculture, and burning, are transported through water systems as suspended particulate matter, dissolved ions, or colloids [59,60]. This suspended matter is continuously redistributed by seasonal floods, facilitating cattle exposure to toxic metals from multiple origins. The second component (PC2) was dominated by Hg, indicating a distinct contamination pattern in samples from Itaituba, located in the Tapajós River basin. This region is widely known for its chronic Hg levels, resulting from both natural sources [61,62] and anthropogenic gold mining activities [63,64]. Seasonal flooding enhances Hg remobilization, particularly under reducing conditions, favoring methylation and increasing its bioavailability in aquatic plants and grasses, which cattle consume.

These findings highlight the central role of Amazonian fluvial systems, particularly the Amazon and Tapajós rivers, not only as natural carriers of trace elements but also as modulators of environmental contaminant exposure in livestock production areas. The identification of distinct bioaccumulation patterns among the studied municipalities (see Figure 2) highlights the importance of environmental and health surveillance, showing that different combinations of geological, hydrological, and anthropogenic factors can shape regional contamination profiles. 

In Iran [65] heavy metals in muscle tissues of cattle submitted to a polluted environment did not present bioaccumulation of Cd and Hg, however, the mean Pb were above the MRL-EU. Similar result of high Pb values was found in Ethiopia by Akele [66] in bovine muscle living in pollution-prone environments. In Brazil, studies on heavy metals in cattle points to increases only in cases of acute intoxication [67], with limited research on chronic exposure and human health risks, especially in Amazon [68].

The EDI of bovine muscle for the elements As, Cd, Hg and Pb, revealed values below the established limits (RfDo). Beef is among the three most consumed proteins in the Amazon region, alongside fish and chicken. Although there is limited data of risk assessment for cattle, studies on the risk of fish consumption have been carried out in various aquatic environments known to be subjected to activities that emit potential toxic metals, such as in the Aegean Sea [69] and Caspian Sea [70], revealing an EDI above the established limits and classified as having serious risks to human health with potential carcinogenic risk. In the Amazon region, the EDI for As, Hg and Pb were above RfDo in almost half of the fish species analyzed and with carcinogenic potential to consumer populations [71].

The results obtained from the determination of the Hazard Quotient showed values lower than 1 (HQ < 1), suggesting that poisoning or adverse effects resulting from the ingestion of each metal in beef are unlikely. The Target Hazard Quotient revealed no exposure (THQ < 1) to harmful effects resulting from the ingestion of these elements. The Total Target Hazard Quotient indicated no combined risk of all toxic metals, with the absence of potential adverse effects. THQ and TTHQ data have been instrumental in verifying populations exposed to toxic metals from food [54,55].

These estimates have already revealed health risks due to fish [72,73,74] and dairy products [75] consumption in Iran, in studies with fruits in Armenia [76], and from groundwater consumption in Cambodia [77]. For the Amazon region, the present study is the first to evaluate human health risks for toxic metals due to beef consumption. A comprehensive review of environmental toxic heavy metals in red meat showed that As and Hg concentration are below standard limits, but Pb and Cd exceed maximum levels, results different that we observed for cattle in western Pará [78]. A large study with toxic metals (As, Cd, and Pb, but not Hg) in cattle from several cities in Brazil showed only liver and kidney samples with concentration above MRL, but none in Pará State [79].

The carcinogenic health risk values associated with the consumption of bovine muscle (see Table 6) showed that both the individual carcinogenic risk (CR) values for arsenic and cadmium, as well as the total carcinogenic risk (TCR), remained below the threshold of concern established at 1.0 × 10^−4^ [49]. The estimated CR values for As were consistently higher than those for Cd across all three evaluated locations, reflecting the higher intrinsic carcinogenic potency of As and positioning it as the main contributor to the total health risk related to beef consumption in the region. Among the municipalities analyzed, Monte Alegre exhibited the highest TCR value, followed by Oriximiná and Itaituba, suggesting spatial variations in exposure. Despite these differences, all estimated TCR values remained within the range considered safe, indicating that, under the specific conditions of this study, the consumption of beef does not pose a significant cancer risk to the local exposed population.

This study is the first to assess the risks associated with ingestion of toxic metals through cattle meat consumption in the Amazon region. Our findings suggest that cattle may serve as bioindicators of environmental exposure to potentially toxic elements, which could pose health risks to Amazonian populations. Future research focusing on discarded female cattle, typically exposed to pasture environments for over eight years, may further clarify the species’ potential as indicators of long-term environmental contamination. In addition, paired environmental samples (soil, water, forage) from cattle´s farm and seasonal sampling would provide information that may elucidate the traceability of contamination pathways. 

Although the overall assessment indicates no significant non-carcinogenic health risk from As, Cd, Hg, and Pb in cattle meat from Monte Alegre, Itaituba, and Oriximiná, the detection of elevated Hg levels in 10% of the samples warrants concern. Notably, each city had at least one animal with muscle Hg concentrations exceeding the EU maximum residue limit (0.01 mg/kg wet weight). These findings suggest localized contamination and highlight the potential for bioaccumulation of mercury. Given the increasing anthropogenic activities in the region, such as mining and deforestation, ongoing surveillance of heavy metal levels in animal tissues is critical to ensure food safety and public health [80,81].

## 5. Conclusions

Cattle meat from Monte Alegre, Itaituba, and Oriximiná poses no significant carcinogenic health risk from As or Cd, nor non-carcinogenic health risk from As, Cd, Hg, and Pb, either individually or combined (TTHQ < 1). Nonetheless, the detection of Hg concentrations above the EU MRL in 10% of the samples across all three cities suggest localized contamination. These results underscore the need for ongoing monitoring of heavy metal residues in animal tissues, particularly given the rising anthropogenic pollution in the western Pará.

## Figures and Tables

**Table 1:** Concentrations of toxic elements (expressed in mg/kg wet weight) in muscle of cattle from Western Pará, Lower Amazonas, Brazil.

Cities	Metal Concentration in Muscle Tissue (mg/kg)			
	As	Cd	Hg	Pb
	Mean ± SEM (Range)	Mean ± SEM (Range)	Mean ± SEM (Range)	Mean ± SEM (Range)
Itaituba	0.01 ± 0.001 (0.004–0.013)	0.004 ± 0.001 (0.0004–0.014)	0.01 ± 0.01 (0.003–0.035)	0.04 ± 0.01 (0.011–0.125)
Monte Alegre	0.009 ± 0.01 (0.0003–0.059)	0.011 ± 0.01 (0.001–0.031)	0.004 ± 0.001 (0.001–0.011)	0.07 ± 0.03 (0.011–0.242)
Oriximiná	0.006 ± 0.002 (0.001–0.019)	0.015 ± 0.004 (0.001–0.037)	0.005 ± 0.001 (0.001–0.014)	0.02 ± 0.004 (0.004–0.043)
MRL Brazil ^α^	0.5	0.05	0.03	0.1
MRL EU ^β^	0.5	0.05	0.01	0.1

MRL: maximum recommended level: ^α^ Anvisa (Normative Instruction, nº 88/2021. ^β^ European Union, commission regulation (EC) nº 466/2001.

**Table 2:** Principal Component Analysis (PCA) loading of toxic metals in cattle muscle from Western Pará.

Metals	PC 1	PC 2
As	0.65037	−0.00361
Cd	0.43893	0.51714
Hg	−0.13484	0.83566
Pb	0.60513	−0.18501

**Table 3:** Estimated daily intake (mg/kg person/day) of toxic metals.

City	Metal			
	As	Cd	Hg	Pb
Itaituba	0.0000071	0.00000406	0.00000913	0.0000375
Monte Alegre	0.00000913	0.0000112	0.00000406	0.0000669
Oriximiná	0.00000609	0.0000152	0.00000507	0.0000223
RfD	0.0003	0.001	0.005	0.004

**Table 4:** Risk quotient (HQ) for toxic metals evaluated in cattle muscles for consumption in the western region of Pará State.

City	HQ			
	As	Cd	Hg	Pb
Itaituba	0.020	0.004	0.020	0.009
Monte Alegre	0.030	0.011	0.008	0.017
Oriximiná	0.020	0.015	0.010	0.006

**Table 5:** Target risk coefficient (THQ) and target total risk quotient (TTHQ) in beef (expressed in mg/kg wet weight) for the scenario of the western region of Pará State.

City	THQ				TTHQ
	As	Cd	Hg	Pb	
Itaituba	0.02	0.004	0.02	0.01	0.06
Monte Alegre	0.03	0.01	0.01	0.017	0.07
Oriximiná	0.02	0.02	0.01	0.006	0.02

**Table 6:** Individual cancer risk (CR) for As and Cd and total cancer risk (TCR) for the combined metals due the ingestion of cattle meat from the western region of Pará State.

City	CR		TCR
	As	Cd	
Itaituba	0.11 × 10^−4^	0.015 × 10^−4^	0.12 × 10^−4^
Monte Alegre	0.14 × 10^−4^	0.04 × 10^−4^	0.18 × 10^−4^
Oriximiná	0.09 × 10^−4^	0.06 × 10^−4^	0.15 × 10^−4^

## Data Availability

The data supporting the results of this study is available upon request.
